# Antioxidants, Oxidative Stress, and Non-Communicable Diseases

**DOI:** 10.3390/antiox11061080

**Published:** 2022-05-29

**Authors:** Luís Crisóstomo, Pedro F. Oliveira, Marco G. Alves

**Affiliations:** 1 Departamento de Anatomia, e Unidade Multidisciplinar de Investigação em Biomedicina (UMIB), Instituto de Ciências Biomédicas Abel Salazar (ICBAS), Universidade do Porto, 4050-313 Porto, Portugal; luis.d.m.crisostomo@gmail.com; 2 Laboratory for Integrative and Translational Research in Population Health (ITR), University of Porto, 4050-600 Porto, Portugal; 3 Integrative Physiology and Pharmacology Unit, Institute of Biomedicine, University of Turku, Kiinamyllynkatu 10, 20520 Turku, Finland; 4 QOPNA & LAQV, Department of Chemistry, University of Aveiro, Campus Universitário de Santiago, 3810-193 Aveiro, Portugal; p.foliveira@ua.pt; 5 Biotechnology of Animal and Human Reproduction (TechnoSperm), Institute of Food and Agricultural Technology, University of Girona, ES-17003 Girona, Spain; 6 Unit of Cell Biology, Department of Biology, Faculty of Sciences, University of Girona, ES-17003 Girona, Spain

Non-communicable diseases have become the leading cause of death, morbidity, and loss of healthy years worldwide, according to the World Health Organization [1]. This trend has not changed even after the COVID-19 pandemic, which rapidly spread across the world, hitting countries with well-established health systems and countries where medical care is not widely available equally. According to preliminary data, mortality attributed to non-communicable diseases was higher than the mortality rate of COVID-19 over the past two years [2]. Non-communicable diseases comprise a large spectrum of diseases, including coronary diseases, the majority of cancers, allergic diseases, diabetes, and even infertility.

The etiology of non-communicable diseases is as diverse as the spectrum of diseases classified as such. Oxidative stress is a transversal phenomenon in aerobic systems, and it is often observed as a manifestation of a pathologic state. Thus, oxidative stress has been implicated with the etiology of several diseases, whereas antioxidant therapy has been suggested to treat disease or alleviate symptoms, particularly in chronic diseases [3].

The growing interest in the potential applications of antioxidant therapy in the context of chronic diseases is well-documented in the three review papers included in this Special Issue on “Antioxidants, Oxidative Stress, and Non-communicable Diseases” [4,5,6]. Han et al. explored the molecular basis of allergic rhinitis, suggesting an oxidative stress basis for this chronic, impairing disease [4]. Allergic rhinitis is closely associated with asthma, and there is therapy to curb the symptoms of both diseases. However, a significant proportion of patients are irresponsive to these therapies, especially adults. Antioxidant-based therapies may be used to complement traditional therapies to improve their efficacy and avoid long-term loss of effect [4].

The complementarity between traditional pharmacological therapies and antioxidants is further explored by Tsai et al. [5]. In this review, the authors highlighted the antioxidant properties of the sodium-glucose cotransporter 2 (SGLT2) inhibitors, a new class of antidiabetic drugs. Besides the effects on glycemic control, SGLT2 inhibitors have shown positive effects in other diseases, by their effect on redox homeostasis [5].

Aging is another phenomenon related to oxidative stress and a risk factor for the onset of non-communicable diseases. Leisegang et al. discussed the role of age-related oxidative stress in the onset of male secondary hypogonadism, i.e., hypogonadism associated with the endocrine disruption caused by non-communicable diseases [6]. The authors highlighted the potential benefits of oxidative management in these patients, notably through lifestyle intervention, hormone replacement therapy, and antioxidant supplementation [6].

The effects of antioxidant supplementation on reproductive health are further explored in two original works in humans, in the context of pregnancy, included in our Special Issue [7,8]. These studies contribute to the still insufficient clinical evidence on the effects of antioxidant supplementation on human health. In their work, Kyozuka et al. retrospectively analyzed pre-conceptional selenium intake and the onset of gestational diabetes (GDM) in a large Japanese cohort [7]. Using multiple logistic regression, the authors estimated an increased risk for late-onset GDM in women with low pre-conceptional selenium intake, and an increased risk for the onset of GDM in women with very high selenium intake [7]. These results show that selenium can protect pregnant women from GDM, but its supplementation must be dosed carefully.

The protective role of antioxidants against the onset of GDM is also the study object of the randomized controlled trial by Basu et al. [8]. The authors recruited 34 obese pregnant women to start an 18-week supplementation program consisting of whole blueberries and fibers, or control. The results suggest an increase in serum total glutathione and antioxidant activity after the supplementation program, compared with the group who received the placebo, and a decrease in serum malondialdehyde, an oxidative stress marker [8].

Excess adiposity and obesity are often associated with oxidative stress. Gusti et al. inverted this paradigm and investigated the prevalence of mutations of the main antioxidant enzymes—superoxide dehydrogenase (SOD), catalase (CAT), glutathione peroxidase (GPX), glutathione-S-transferase (GST), and nitric oxide synthase (NOS)—in a Saudi obese population [9]. Of the 12 Single nucleotide polymorphisms (SNPs) selected by the authors, 4 gene variants were related to an increased risk of obesity in the studied population [9].

Oxidative stress is often a marker of an active inflammatory process. Based on this premise, Silva-Vaz et al. quantified serum oxidative stress, inflammatory biomarkers, and metabolites and related those parameters with the staging of biliary acute pancreatitis (AP) in a Portuguese cohort [10]. The authors found differences in serum phenylalanine, threonine, and lipids between patients with different stages of AP, hence illustrating the potential role of these metabolites as biomarkers of AP progression. Interestingly, the authors reported differences in the markers of oxidative stress between AP patients and healthy controls, suggesting a role of oxidative stress in the onset of the disease [10].

The role of oxidative stress in cancer has been studied to develop new therapies to inhibit the growth and progression of cancer cells, but also alleviate the symptoms of the disease. Krystek-Korpacka et al. monitored serum and erythrocyte antioxidant defenses in a Polish cohort of colorectal cancer patients, during the early postoperative period, to correlate oxidative stress parameters with clinical outcomes [11]. Based on their data, the authors identified the period between 8 h and 24 h after surgery as the most sensitive period for oxidative homeostasis and positive health outcomes.

Ivermectin, a drug used in parasitic infections, has recently been linked to disinformation related to its efficacy against SARS-CoV-2. Zhang et al. raised further awareness against self-medication and unsupervised use of ivermectin beyond the scope of its primary application, with an in vitro study linking this drug to neurotoxicity [12]. In this toxicological study, human neuroblastoma-derived SH-SY5Y cells were treated with increased doses of ivermectin, resulting in increased production of reactive oxygen species, mitochondrial dysfunction, and autophagy. The authors further identified the inhibition of the Akt–mTOR pathway as the cause of the apoptotic and autophagic events in this cell line [12].

Antioxidant therapy remains a promising strategy, but more studies are needed to overcome the main barriers to the implementation of these innovative therapies, notably the specificity, effectivity, and directed delivery of antioxidants. Despite that, “Antioxidants, Oxidative Stress and Non-communicable Diseases” continues to be a growing research topic, as supported by the variety and novelty of the works included in this Special Issue, from in vitro studies to clinical practice.
